# CK1ε drives osteogenic differentiation of bone marrow mesenchymal stem cells via activating Wnt/β-catenin pathway

**DOI:** 10.18632/aging.205067

**Published:** 2023-10-02

**Authors:** Zhentang Yu, Xijia Jiang, Jianjian Yin, Lei Han, Chengwei Xiong, Zhennan Huo, Jie Xu, Jingjing Shang, Kun Xi, Luming Nong, Yong Huang, Xindie Zhou

**Affiliations:** 1 Department of Orthopedics, The Affiliated Changzhou Second People’s Hospital of Nanjing Medical University, Changzhou 213000, China; 2 Department of Orthopedics, Yibin Integrated Traditional Chinese and Western Medicine Hospital, Yibin 644104, China; 3 Department of Graduate School, Dalian Medical University, Dalian 116000, China; 4 Department of Pharmacy, The Affiliated Changzhou Second People’s Hospital of Nanjing Medical University, Changzhou 213000, China; 5 Department of Orthopedics, The First Affiliated Hospital of Soochow University, Orthopedic Institute, Soochow University, Suzhou 215006, China; 6 Department of Orthopedics, Gonghe County Hospital of Traditional Chinese Medicine, Hainan Tibetan Autonomous Prefecture, Qinghai 811800, China; 7 Changzhou Medical Center, Nanjing Medical University, Changzhou 213000, China

**Keywords:** bone defect, bone regeneration, osteogenic differentiation, mesenchymal stem cells, Wnt/β-catenin, CK1ε

## Abstract

The treatment of bone defects is a difficult problem in orthopedics. At present, the treatment mainly relies on autologous or allogeneic bone transplantation, which may lead to some complications such as foreign body rejection, local infection, pain, or numbness at the bone donor site. Local injection of conservative therapy to treat bone defects is one of the research hotspots at present. Bone marrow mesenchymal stem cells (BMSCs) can self-renew, significantly proliferate, and differentiate into various types of cells. Although it has been reported that CK1ε could mediate the Wnt/β-catenin pathway, leading to the development of the diseases, whether CK1ε plays a role in bone regeneration through the Wnt/β-catenin pathway has rarely been reported. The purpose of this study was to investigate whether CK1ε was involved in the osteogenic differentiation (OD) of BMSCs through the Wnt/β-catenin pathway and explore the mechanism. We used quantitative reverse transcription-polymerase chain reaction (qRT-qPCR), Western blots, immunofluorescence, alkaline phosphatase, and alizarin red staining to detect the effect of CK1ε on the OD of BMSCs and the Wnt/β-catenin signaling pathway. CK1ε was highly expressed in BMSCs with OD, and our study further demonstrated that CK1ε might promote the OD of BMSCs by activating DLV2 phosphorylation, initiating Wnt signaling downstream, and activating β-catenin nuclear transfer. In addition, by locally injecting a CK1ε-carrying adeno-associated virus (AAV5- CK1ε) into a femoral condyle defect rat model, the overexpression of CK1ε significantly promoted bone repair. Our data show that CK1ε was involved in the regulation of OD by mediating Wnt/β-catenin. This may provide a new strategy for the treatment of bone defects.

## INTRODUCTION

Bone defects are one of the most complicated problems in clinical orthopedics. It can be caused by many factors such as trauma, infection, and tumor resection [1]. The treatment of bone defects usually requires extensive autologous or allogeneic bone grafting and reconstruction, while the treatment often brings complications such as foreign body rejection, local infection, pain, and numbness at the bone donor site [2]. For large bone defects, the effect of conservative treatment is very limited. The process of bone healing or regeneration is a continuous event involving various molecules and proteins [3, 4]. Early local chronic inflammation starts the innate immunity and vascular permeability of the body, and inflammatory cells such as macrophages and neutrophils are recruited. Subsequently, the secretion of corresponding inflammatory factors and cytokines promotes the homing of bone marrow mesenchymal stem cells (BMSCs) [5]; then the withdrawal of inflammatory cells and the secretion of TGF-β, IL-10, and other inflammatory factors from macrophages promote the OD of mesenchymal stem cells [2, 6, 7]. During the whole process, BMSCs play an important role as the cornerstone and seed cells. At present, in addition to standard treatments such as bone transplantation, the research on drugs and materials for bone regeneration and repair materials based on stem cells has also shown new vitality [8, 9].

BMSC is a subgroup of non-hematopoietic stromal cells residing in bone marrow, capable of self-renewal and differentiation into multiple cell types, as well as the ability to self-renew and significantly proliferate [10]. BMSCs have become the most widely studied bone regenerative mesenchymal cells due to their close association with bone pathophysiology. The balance between osteogenic differentiation (OD) and osteoclast differentiation is an important mechanism for the occurrence and development of bone homeostasis. At the cellular level, BMSCs strongly regulate bone formation and resorption by differentiating into osteoblasts and modulating osteoclast activity [11]. Pathologically, BMSC dysfunction has been revealed to be a key cellular mechanism in various bone diseases, including bone defects [12]. Importantly, BMSCs, as effective microenvironmental regulators, play a significant anti-inflammatory role after transplantation, benefiting various tissues/organs including bone [13]. According to various studies, BMSCs have good therapeutic potential in bone defects through cell therapy or tissue engineering [14, 15]. Studies on the regulation of BMSC lineage differentiation have attracted increasing attention [16]. The differentiation direction of BMSCs is regulated by chemical, physical, and biological factors. These factors trigger different signaling pathways and activate multiple transcription factors that guide BMSC participation in a lineage [17].

Wnt signaling is the main evolutionarily-conserved signaling pathway that controls embryonic development and adult tissue homeostasis. The Wnt/β-catenin pathway, the classical Wnt signaling pathway, plays an important role in regulating OD, and the formation of bone matrix and mineralization [18, 19]. Dysregulation in Wnt signaling leads to serious bone-related disorders such as osteoporosis and osteosclerosis [20]. Some studies [21–23] have showed that CK1 mediated the Wnt/β-catenin pathway. The serine (Ser)/threonine (Thr) protein kinases of the casein kinase 1 (CK1) family participate in the development of cells. CK1 can participate in the regulation of the Wnt signal transduction cascade from the membrane receiving system to transcriptional effector [24]. CK1 genes form a unique and small branch of the protein kinase family. Six CK1 isoforms are encoded by distinct genes (α1, δ, ε, γ1, γ2, and γ3), which are post-transcriptionally processed to yield a number of splice variants [25]. Our previous study found that CK1ε, not CK1α1, CK1δ, CK1γ1, CK1γ2, or CK1γ3, showed abnormal expression during the OD of BMSCs (Supplementary Figure 1). Recent studies showed that CK1ε played a key role in regulating human health conditions such as day-night rhythms, tumors, and diabetes [18, 26]. Foldynová-Trantírková et al. [27] showed that mutations in CK1ε not only suppressed Wnt/β-catenin, but also stimulated the Wnt/Rac-1/JNK and Wnt/NFAT pathways, thereby promoting the development of breast cancer by affecting cell adhesion and migration. They considered that a point mutation in the CK1εN terminal lobe in breast cancer was related to a decrease in the phosphorylation activity of CK1ε *in vitro* and *in vivo*. Qiong et al. [28] reported that transforming growth factor (TGF)-β1 induced CK1ε activation to promote β-catenin nuclear accumulation, which then regulated chondrogenesis-related gene transcription to eventually promote mouse precartilaginous stem cell differentiation. However, whether CK1ε has a similar effect on BMSC differentiation has not been reported. Based on the above theories and previous research results, we supposed that CK1ε may accelerate the OD process in BMSCs by mediating the Wnt/β-catenin pathway, thereby promoting bone regeneration. However, the definitive mechanism remains to be studied. Therefore, this study was undertaken to reveal the role of CK1ε in the molecular regulation mechanism of the OD of BMSCs to provide new therapeutic agents for bone defect treatment.

## MATERIALS AND METHODS

### Acquisition of BMSC samples

This experiment selected four 4-week-old SPF grade SD male rats, weighing approximately 300 g, purchased from Changzhou Cavens Experimental Animal Co., Ltd. (Changzhou, China). The animal program has been approved by the Ethics Committee of the First Affiliated Hospital of Soochow University, with an animal ethics approval number of 2022-552. All animal surgical procedures were performed according to the protocols approved by the Ethics Committee of the First Affiliated Hospital of Soochow University. Sprague-Dawley (SD) rats were euthanized by cervical dislocation and soaked in 75% alcohol for 15 min. Bilateral femurs were removed under sterile conditions, and the bone marrow cavity was repeatedly washed by aspirating with Dulbecco’s modified Eagle Medium (DMEM/F12, Shanghai Zhong Qiao Xin Zhou Biotechnology Co., Ltd., Shanghai, China) using a 1-mL sterile syringe, and collected into a centrifuge tube. The bone marrow cell suspensions were centrifuged. Bone marrow mononuclear cells were removed, washed, and enriched for BMSCs by culture in a culture bottle containing DMEM/F12 and 10% fetal bovine serum (FBS, Gibco, Waltham, MA, USA) at 37°C and 5% CO_2_. Cells collected from a rat were cultured in a culture bottle. The culture medium was replaced every other day until reaching 80–90% cell confluence. These subcultured cells were designated as the first passage (P1), and P3 was used for subsequent experiments.

### Cell culture and transfection

Routine OD induction culture procedure: BMSCs were grown in DMEM/F12 containing 10% FBS, 50 ug/mL of streptomycin, and 100 U/mL of penicillin at 37°C and 5% CO_2_ until reaching 70–80% cell confluence. Then, the initial culture medium was removed, and an osteogenic induction culture medium (OIM, RAXMX-90021, Cyagen, Taicang, China. Ingredients: OriCell^®^Basal medium for cell culture, 177 mL; OriCell^®^Fetal bovine serum (Superior-Quality), 20 mL; OriCell^®^Supplement for rat bone marrow mesenchymal stem cells OD, 3 mL; alizarin red stain, 10 mL; Gelatin, 10 mL) was added. Si-NC (20 nM), CK1ε siRNAs (20 nM), pcDNA3.1 (2 μg/mL), and pcDNA3.1-CK1ε (2 μg/mL) were purchased from Tsingke Biotechnology Co., Ltd. (Beijing, China). Due to the time of osteogenic induction and the aging of transfection, OD induction was initiated 24 hours after transfection, and transfection was performed again on the 5th and 10th day, respectively, to maintain the efficiency of transfection. The proliferation efficiency and morphology of BMSCs did not change significantly after the first transfection. 24 h after transfection, OD culture began. At this time, the proliferation efficiency of BMSCs began to decline, and the morphology of some cells gradually changed from spindle to polygon. After the second and third transfection and OD induction culture, the cell proliferation efficiency was further reduced, and the cell morphology was completely polygon. 7 days later, nodular structures gradually began to appear in the cultured cells. In addition, DKK-1 (Wnt inhibitor; MCE, Monmouth Junction, NJ, USA) at a concentration of 0.5 μg/mL and pcDNA3.1-CK1ε were co-transfected to verify the interaction between CK1ε and Wnt/β-catenin. Transfections were carried out using lipofectamine 2000 (Invitrogen, Waltham, MA, USA) [29]. IWP-O1 (DVL2 phosphorylation inhibitor, MCE) at a concentration of 1 uM was co-cultured to explore the specific relationship between CK1ε and DVL2.

### Alizarin red staining (ARS)

After exposure to OIM for two weeks, the medium was removed. The BMSCs were washed with phosphate-buffered saline (PBS) twice and then fixed in 4% paraformaldehyde for 30 min. BMSCs were washed with double-distilled water and stained with 0.2% alizarin red stain (Leagene, Guangzhou, China) for 30 minutes at 37°C. The staining results were observed under a microscope [19]. For quantifying the relative amount of calcium, 400 μL 10% (w/v) cetylpyridinium chloride (CPC; Sigma-Aldrich, St. Louis, MO, USA) and 10 mM sodium phosphate solution were added to the stained dishes, and the absorbance of the extracted dye was determined at a wavelength of 562 nm.

### Determination of cellular calcium content

Calcium concentrations were measured using a calcium colorimetric assay kit (S1063S, Beyotime Biotechnology, Jiangsu, China), according to the manufacturer’s instructions. Briefly, the cells were washed with PBS and suspended in buffer. Working solution (150 μL) was added to each well and the cells were incubated at 37°C for 5 min. Absorbance at 575 nm was measured using a SpectraMax M2 spectrometer (Molecular Devices, Sunnyvale, CA, USA) [30, 31].

### Quantitative reverse transcription-polymerase chain reaction (qRT-PCR) analysis

After the osteogenic induction culture of BMSCs for 14 days, total RNA was extracted using TRIzol (Takara, Beijing, China). Osteoblast-related genes RUNX2, OCN, SP7, COL1A1, CK1ε, β-catenin, and GAPDH were assayed using the mRNA PCR Detection Kit (Takara). The 2^−ΔΔCt^ method [32] was used for quantitatively analyzing the results. GAPDH was used as the internal reference in this experiment. The primer sequences (Invitrogen) are shown in Table 1.

**Table 1:** Primer sequences used in this research.

**Gene names**	**Forward sequence (5′–3′)**	**Reverse sequence (5′–3′)**
RUNX2	CGCCTCACAAACAACCACAG	AATGACTCGGTTGGTCTCGG
OCN	CACCACCGTTTAGGGCATGT	CTTTCGAGGCAGAGAGAGGG
SP7	TCTAGGATTGGATCTGAGTGAGCC	CATAGTGAGCTTCTTCCTGGGG
COL1A1	GAGACAGGCGAACAAGGTGA	GGGAGACCGTTGAGTCCATC
CK1ε	AGGAAGAGAAGCAGCAGAGC	GTGCTACCCAGAGCCAGTTT
β-catenin	CACCATCGAGAGGGCTTGTT	CTGGCGACCCAAGCATTTTC
GAPDH	TCTCTGCTCCTCCCTGTTCT	ATCCGTTCACACCGACCTTC

### Alkaline phosphatase (ALP) activity assay and staining

BMSCs cultured in different groups for 14 days were collected and lysed. After centrifugation, ALP activity was assayed in the BMSC lysates using a commercial ALP assay kit, (P0321, Beyotime), and the results were read on a microplate reader (Multiskan FC, Thermo Fisher Scientific, Waltham, MA, USA) [18]. BMSCs were also stained with an ALP Stain Kit (G1480, Kaplow’s/Azo Coupling Method, Solarbio, Beijing, China) and observed under a microscope.

### Western blot analysis

The total proteins in the BMSCs of the different groups were lysed using RIPA lysis buffer and extracted. The bicinchoninic acid (BCA) protein assay kit (P0010S, Beyotime) was used for measuring protein concentrations. Each protein sample underwent sodium dodecyl sulfate-polyacrylamide gel electrophoresis (SDS-PAGE). After electrophoresis, the proteins were transferred to a polyvinylidene difluoride (PVDF) membrane and washed with TBST. The membranes were incubated with primary antibodies overnight at 4°C and then with horseradish peroxidase-conjugated secondary antibodies for two hours at 37°C [29]. Finally, the proteins were visualized by chemiluminescence autoradiography (Thermo Fisher Scientific). Primary antibodies against RUNX2 (ab76956, Abcam, Waltham, MA, USA), OCN (sc-390877, Santa Cruz, Dallas, TX, USA), COL1A1 (sc-293182, Santa Cruz), SP7 (ab209484, Abcam), CK1ε (ab302638, Abcam, and sc-373912, Santa Cruz), β-catenin (ab68183, Abcam), C-JUN (ab40766, Abcam), C-MYC (ab32072, Abcam), LEF1 (ab137872, Abcam), TCF7 (C.725.7, Invitrogen, USA), disheveled 2 (DVL2) (phospho T224, ab124941, Abcam), and DVL2 (ab228804, Abcam) were used in this experiment, and GAPDH (ab8245, Abcam) was used as the internal reference [29, 33].

### Flow cytometry

Since mature BMSCs do not express CD11b but express CD44, both CD44 and CD11b were analyzed by flow cytometry using CD44-fluorescein isothiocyanate (FITC) and CD11b-FITC antibodies (BD Biosciences, San Jose, CA, USA) according to the manufacturer’s protocol [34].

### Immunofluorescence staining

After two weeks of incubation, BMSCs in the different groups were fixed in 4% paraformaldehyde for 15 min at room temperature, then permeabilized in 0.1% Triton X for 20 min. To block nonspecific antibody binding sites, the BMSCs were incubated with 1% bovine serum albumin (BSA) for 20 min, then with primary anti-β-catenin antibodies (1: 200) overnight at 4°C for eight hours. The cells were washed three times with PBS and incubated with cy3-labeled rabbit secondary antibody (Jackson, West Grove, PA, USA) diluted 1: 500 for one hour. To observe β-catenin nuclear translocation by fluorescence microscopy, the cells were stained with 4′-6-diamidino-2-phenylindole (DAPI, Invitrogen, USA). Finally, images were obtained on image management software [35].

### Animals

SD rats were purchased from Changzhou Cavens Experimental Animal Co., Ltd. All rats were males, 8–10 weeks old, and weighed about 300 g to 350 g. All surgical procedures and perioperative management complied with Chinese laws and the regulations of the local ethics committee.

### Establishment of the rat femoral condyle defect model

The rats were randomly divided into four groups: normal group (sham-operation; normal saline treatment, *n* = 10), control group (surgery; normal saline treatment, *n* = 10), AAV5 group (surgery; 100 μL normal saline with 1 × 10^9^ PFU adeno-associated virus 5 (AAV5) lentivirus vector treatment, *n* = 10), and AAV5-CK1ε group (surgery; 100 μL normal saline with 1 × 10^9^ PFU AAV5 lentivirus vector of CK1ε treatment, *n* = 10). AAV5 lentivirus vector was purchased from OBiO Technology (Shanghai, China) [36]. The rat femoral condyle defect model was used to evaluate the bone repair ability of CK1ε *in vivo*. First, a 2% sodium pentobarbital solution was injected intraperitoneally at a dose of 2.5 mL/kg. The femoral condyle was exposed by carefully and passively separating the muscle space to avoid damaging the surrounding blood vessels and soft tissue. Bicortical perforation was performed with a standard 3-mm drill at low speed, and normal saline was injected at the same time to avoid bone necrosis caused by high temperatures. Finally, to verify the potential target for minimally invasive injection therapy, the muscles, fascia, and skin of the rats were sutured layer by layer, and the AAV5 lentivirus vector was injected through the skin. After surgery, penicillin was routinely injected intramuscularly to prevent infection. The animals were kept at 25°C and 55% humidity, with 12-hour alternating light and dark cycles. The rats were provided with standard laboratory feed and tap water [37–40].

### Specimen collection and computed tomography analysis

SD rats were euthanized four weeks after surgery, and femoral specimens were taken and fixed in a 10% formalin solution. First, the specimen was imaged and evaluated. Micro-computed tomography (micro-CT, Bruker SkyScan 1176, Bruker, Berlin, Germany) was used to analyze the repair of the femoral defects. The micro-CT parameters were 18 μm, 65 kV, and 385 mA. Scanning parameters were 1 mm thick, rotation step 0.7 deg, average 1 frame, sync with event 5 ms and list Mode 2 frame, using SkyScan software. Appropriately 3-mm cylindrical regions were designated as regions of interest using Dataview and CT analyzer software and analyzed for bone mineral density (BMD), bone volume fraction (BV/TV), trabecular separation (TB.sp) and trabecular thickness of bone (TB.th).

### Histological analysis

Decalcified specimens were scanned by micro-CT after incubating in xylene solution at room temperature, gradient dehydration in ethanol, and embedding in paraffin. The embedded femurs were sectioned sagittal to a thickness of 5 μm using a microtome. Tissue sections were stained with hematoxylin and eosin (H&E) and Masson’s stain to analyze tissue regeneration in the defect areas. CD31 immunofluorescence staining (GB113151, Servicebio, Wuhan, China), CK1ε, osteocalcin (23418-1-AP, Proteintech, Rosemont, IL, USA) and osteopontin (OPN, GB11500, Servicebio) immunohistochemical staining were performed on the tissue sections to evaluate angiogenesis and bone repair.

### Statistical analysis

All cell experiments were performed in triplicate. The data were measured three times and expressed as means ± standard deviations (SD). The quantitative analysis of immunohistochemical and immunofluorescence staining was performed on five representative images per group using ImageJ software. The Student’s *t*-test was used to compare the differences between two groups. One-way analysis of variance (ANOVA) was used to compare differences between multiple groups using SPSS 19.0 (SPSS Inc., Chicago, IL, USA) or GraphPad Prism 9.0. A *p*-value of < 0.05 indicated statistical significance.

### Data availability statement

All remaining data are available within the article, or available from the corresponding authors upon request.

## RESULTS

### Identification and related characteristics of BMSCs in mature SD rats

**Figure 1:**
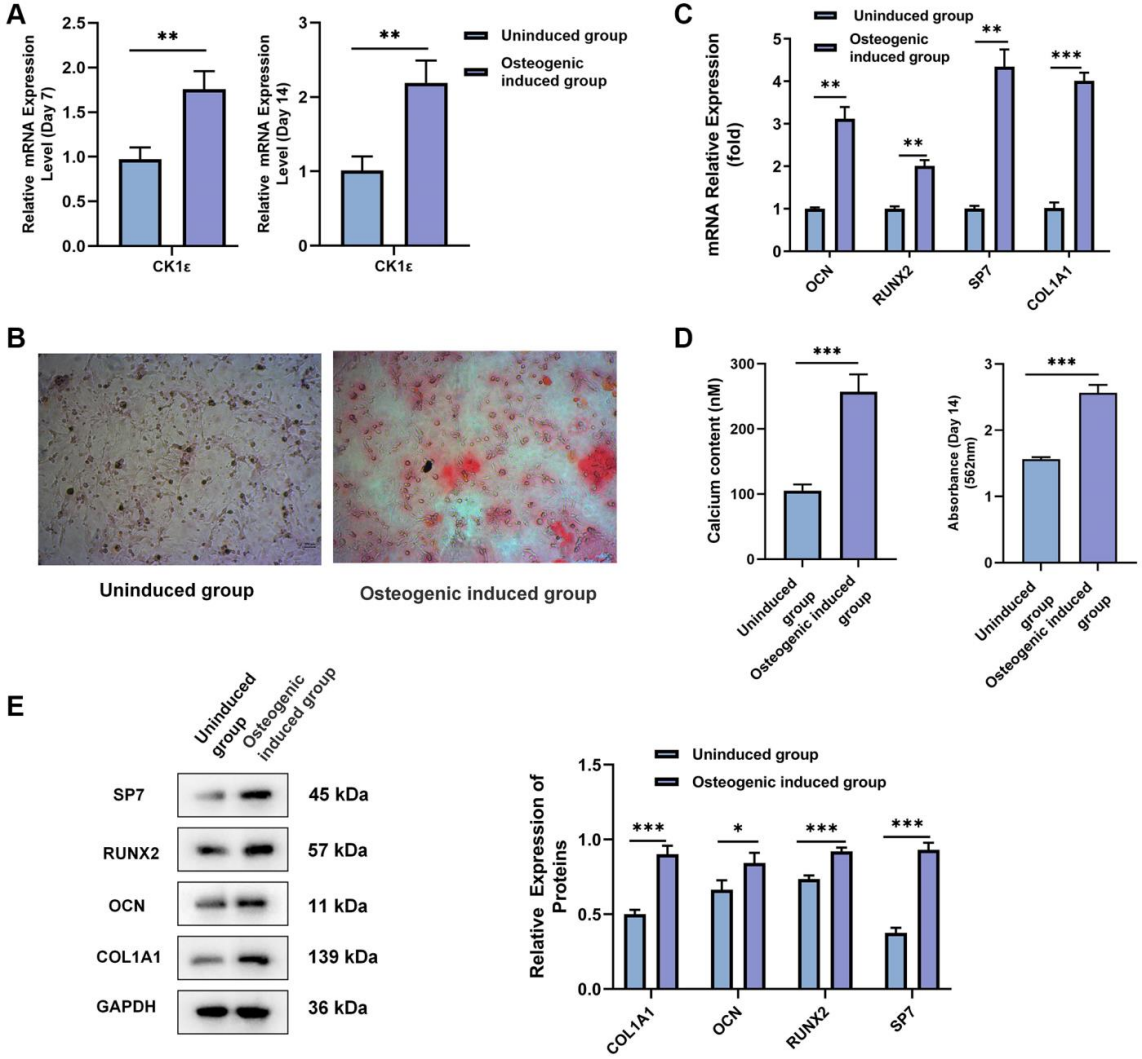
**Identification and confirmation of the OD of rat BMSCs.** (**A**) BMSCs were divided into uninduced and osteogenic induced groups. The relative mRNA expression level of CK1ε was detected by qRT-PCR analysis after 7 and 14 days of incubation. (**B**) ARS of BMSC osteoblasts after two weeks of incubation. (**C**) The relative mRNA expression levels of OCN, RUNX2, SP7, and COL1A1 were detected by qRT-PCR after two weeks of incubation. (**D**) Qualitative analysis for ARS, and intracellular calcium concentration were detected after two weeks of induction. (**E**) Relative protein expression levels and semi-quantitative analysis of SP7, RUNX2, OCN, and COL1A1 after two weeks of incubation. The experiments in this figure were repeated three times, and similar results were obtained. Student’s *t*-test was used for (**A**, **C**, **D**, **E**). The data are presented as the means ± SD of independent experiments. ^*^P < 0.05; ^**^P < 0.01; ^***^P < 0.001; Abbreviation: ns: not statistically significant.

Firstly, BMSCs underwent uninduced or osteogenic induction culture, and follow-up experiments were performed on day 14. Flow cytometry was used to determine the proportion of CD44 and CD11b cells in the osteogenic induced group. As shown in Supplementary Figure 2, 94% of the cells were CD44-positive and CD11b-negative. These results confirmed that these cells isolated from SD rats were mainly BMSCs. Then, qRT-PCR was used to measure CK1ε mRNA expression levels. Compared to the uninduced group, CK1ε relative expression in the osteogenic induced group was significantly higher both on day 7 and 14 (Figure 1A, *P* < 0.01). ARS was used to observe the calcification of the BMSCs after osteogenic induction culture. The results in Figure 1B showed that the staining in the osteogenic induced group was significantly deeper than that in the uninduced group. We subsequently carried out qualitative analysis of the ARS results and further identification of the calcium content in the osteoblasts after induction. As can be seen from the results in Figure 1D, the qualitative analysis results of alizarin red in the osteogenic induced group and the content of calcium in osteoblasts after induction were significantly higher than those in the uninduced group (*P* < 0.001), which indicated that the osteogenic induced group showed a higher degree of calcification after the osteogenic induction culture, which also suggested the success of OD. In order to further verify the success of the OD of the BMSCs, relative mRNA and protein expressions of OD related factors, including RUNX2 [41], OCN [42], SP7 [43], and COL1A1 [44], were detected by qRT-PCR and Western blot. Figure 1C showed that the relative mRNA expression levels of all relevant standard OD markers in the osteogenic induced group were significantly higher than those in the uninduced group (*P* < 0.01). The Western blot gel images and semi-quantitative analysis results further confirmed the higher relative protein expression levels of these related proteins in the osteogenic induced group, with all of the *P* values < 0.05 (Figure 1E).

### Effect of CK1ε on the OD of BMSCs

**Figure 2:**
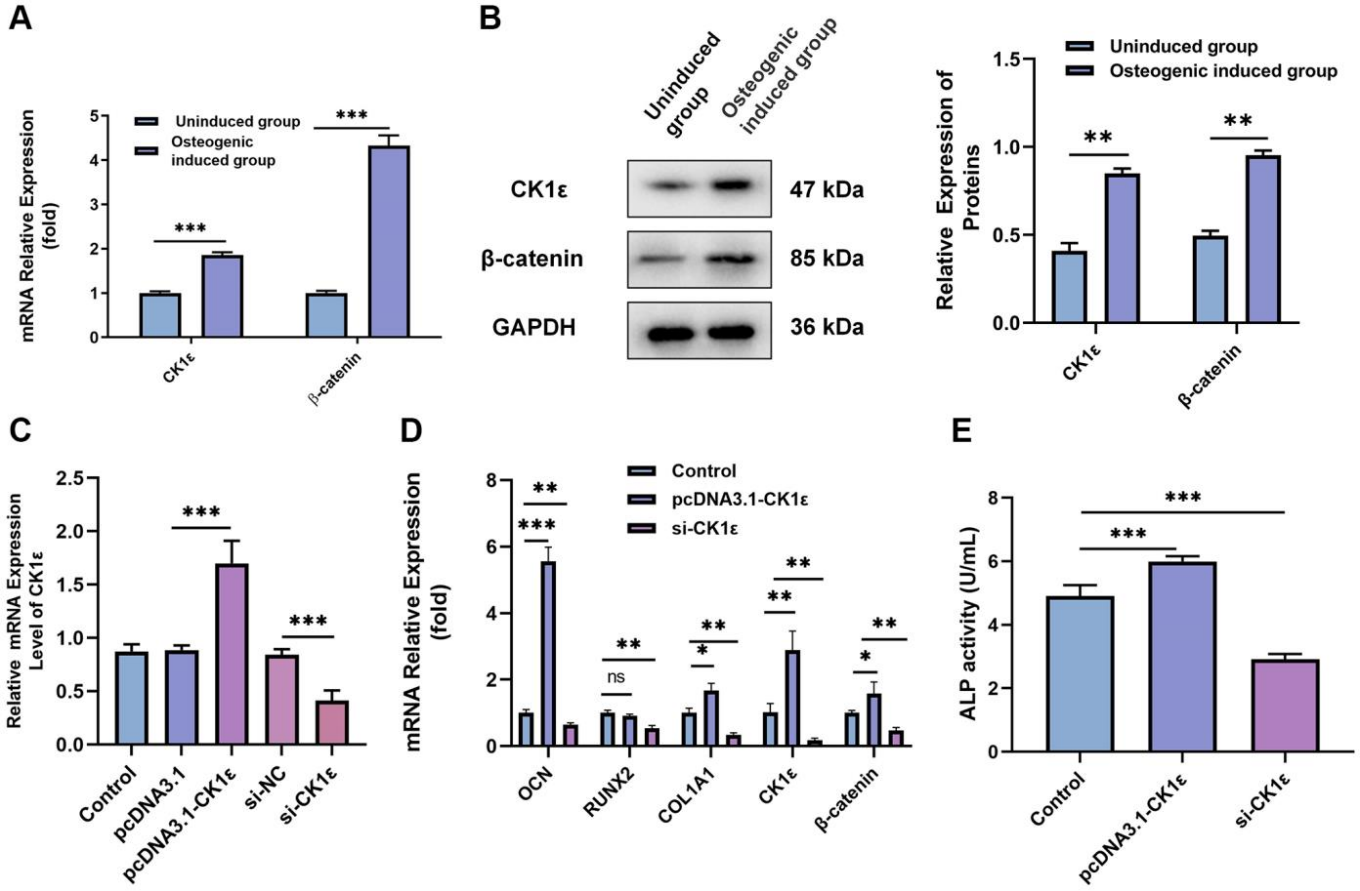
**Relationship between CK1ε, β-catenin, and the OD of BMSCs.** (**A**) Relative mRNA expressions of CK1ε and β-catenin after the OD of BMSC. (**B**) Western blot detection and analysis of relative CK1ε and β-catenin protein levels after two weeks’ induction. (**C**) qRT-PCR was used to detect the relative mRNA expression of CK1ε after transfection. (**D**) The relative mRNA expression levels of OD-related genes (OCN, RUNX2, SP7, and COL1A1) after CK1ε transfection. (**E**) ALP activity on the 14th day of BMSC’s OD with different transfections. The experiments in this figure were repeated three times, and similar results were obtained. The data are presented as the means ± SD of independent experiments. The Student’s *t*-test was used for (**A**, **B**) and one-way ANOVA was used for (**C**–**E**). ^*^P < 0.05; ^**^P < 0.01; ^***^P < 0.001; Abbreviation: ns: not statistically significant.

The role of CK1ε in the OD of BMSCs is the next focus of our study. After two weeks of induction, qRT-PCR and Western blot results showed that CK1ε and β-catenin relative mRNA and protein expressions were up-regulated in the osteogenic induced group when compared with uninduced group (Figure 2A, *P* < 0.001 and Figure 2B, *P* < 0.01). Subsequently, si-CK1ε and pcDNA3.1-CK1ε were constructed and transferred into the BMSCs to down-regulate and up-regulate the expression of CK1ε, respectively. The outcome, as presented in Figure 2C, showed the success of transfection. To explore the role of CK1ε in OD, we designed three groups (control: osteogenic induced group; pcDNA3.1-CK1ε: transfected pcDNA3.1-CK1ε and then induced group; and, and si-CK1ε: transfected si-CK1ε and then induced group) to observe the expression of CK1ε. OD induction was initiated 48 hours after transfection, and transfection was performed again on the 5th and 10th day, respectively, to maintain the efficiency of transfection. All specimens were collected and tested after two weeks’ induction. The qRT-PCR results showed that the relative mRNA expression of OD-related genes (OCN, RUNX2, and COL1A1), CK1ε and β-catenin was significantly up-regulated in the pcDNA3.1-CK1ε group compared with the other two groups (Figure 2D, *P* < 0.05). ALP activity was detected to further verify the OD state and the ability to mineralize. The results further verified that the pcDNA3.1-CK1ε group had higher osteogenic activity, when compared with the other two groups (Figure 2E, *P* < 0.001). Based on the above outcomes, we proposed the hypothesis that the overexpression of CK1ε may promote the OD of BMSCs.

**Figure 3:**
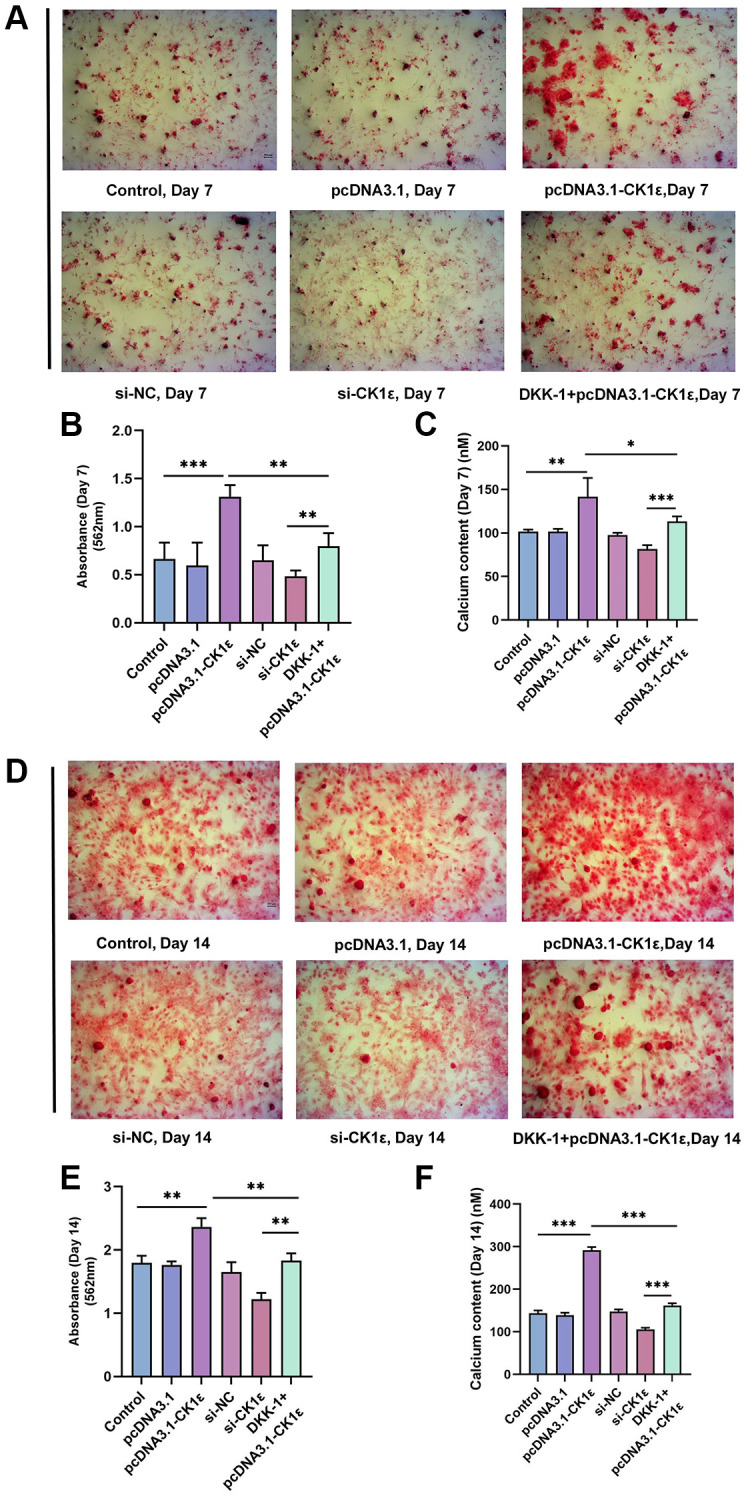
**Detection of mineralization after OD of BMSC.** (**A**) The calcification status of different groups on the 7th day. (**B**) Qualitative analysis of ARS of different groups on the 7th day. (**C**) Detection of intracellular calcium levels after BMSC induction of different groups on the 7th day. (**D**) The calcification status of different groups on the 14th day. (**E**) Qualitative analysis of ARS of different groups on the 14th day. (**F**) Detection of intracellular calcium levels after BMSC induction of different groups on the 14th day. The experiments in this figure were repeated three times, and similar results were obtained. The one-way ANOVA was used for (**B**, **C**, **E**, **F**). ^*^P < 0.05; ^**^P < 0.01; ^***^P < 0.001; Abbreviation: ns: not statistically significant.

**Figure 4:**
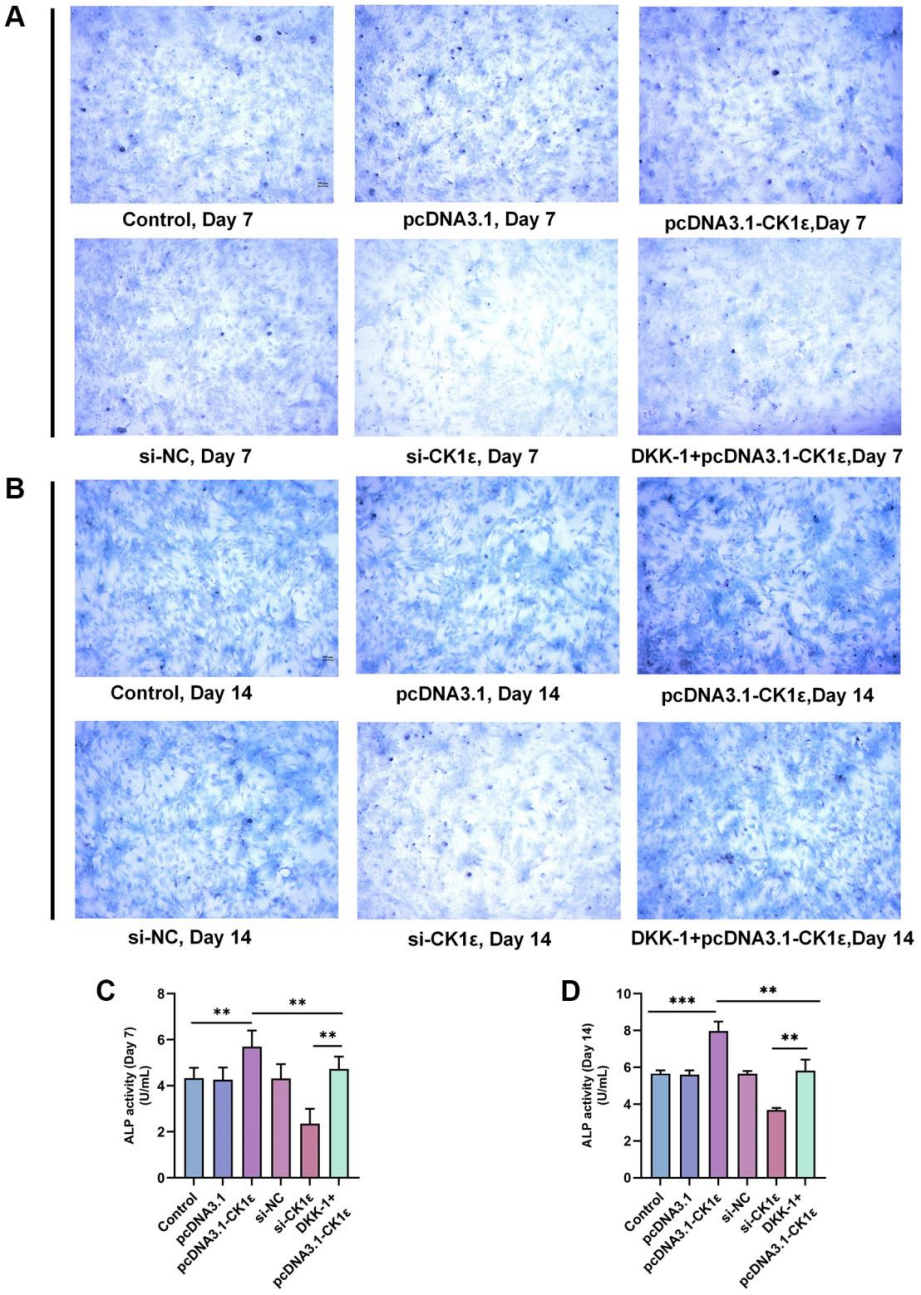
**ALP staining and activity analysis results.** (**A**) Images of ALP staining on the 7th day of BMSC OD with different transfections. (**B**) Images of ALP staining on the 14th day of BMSC OD with different transfections. (**C**, **D**) ALP activity analyses on the 7th and 14th days of BMSC OD with different transfections. The experiments in this figure were repeated three times, and similar results were obtained. The one-way ANOVA was used for (**C**, **D**). ^*^P < 0.05; ^**^P < 0.01; ^***^P < 0.001; Abbreviation: ns: not statistically significant.

Then, we examined the mineralization of different groups. The cells were allowed to grow for two weeks and related events were observed every seven days. Consistent with our hypothesis, the up-regulation of CK1ε significantly increased the calcification state of cells compared to groups with lower or normal expression of CK1ε. The pcDNA3.1-CK1ε group demonstrated the most obvious staining changes on both day 7 and 14, when compared with other groups (Figure 3A, 3D). The results of the ARS qualitative analysis and intracellular calcium concentration confirmed the beneficial role of CK1ε overexpression in promoting OD, and the inhibition effect of low CK1ε expression on OD (Figure 3B, 3C, 3E, 3F, *P* < 0.01). Next, to further study the exact relationship between CK1ε and Wnt/β-catenin, DKK-1 (a Wnt/β-catenin signal pathway inhibitor) was used to interfere with the BMSCs during the whole process of induction. After seven days of incubation, ARS showed reduced calcification levels in the DKK-1+pcDNA3.1-CK1ε group compared with the pcDNA3.1-CK1ε group, which means that blocking the Wnt/β-catenin pathway significantly reduced the level of calcification (Figure 3A–3C, *P* < 0.01). Moreover, the results were more obvious on the 14th day (Figure 3D–3F, *P* < 0.01). Then, we estimated the ALP activity in all the groups. Combined with ALP staining and the activity results, we found that overexpression of CK1ε significantly increased the activity of ALP, while low expression of CK1ε decreased the activity of ALP. Meanwhile, DKK-1 could also reverse the increased ALP activity caused by CK1ε overexpression (Figure 4, *P* < 0.01).

**Figure 5:**
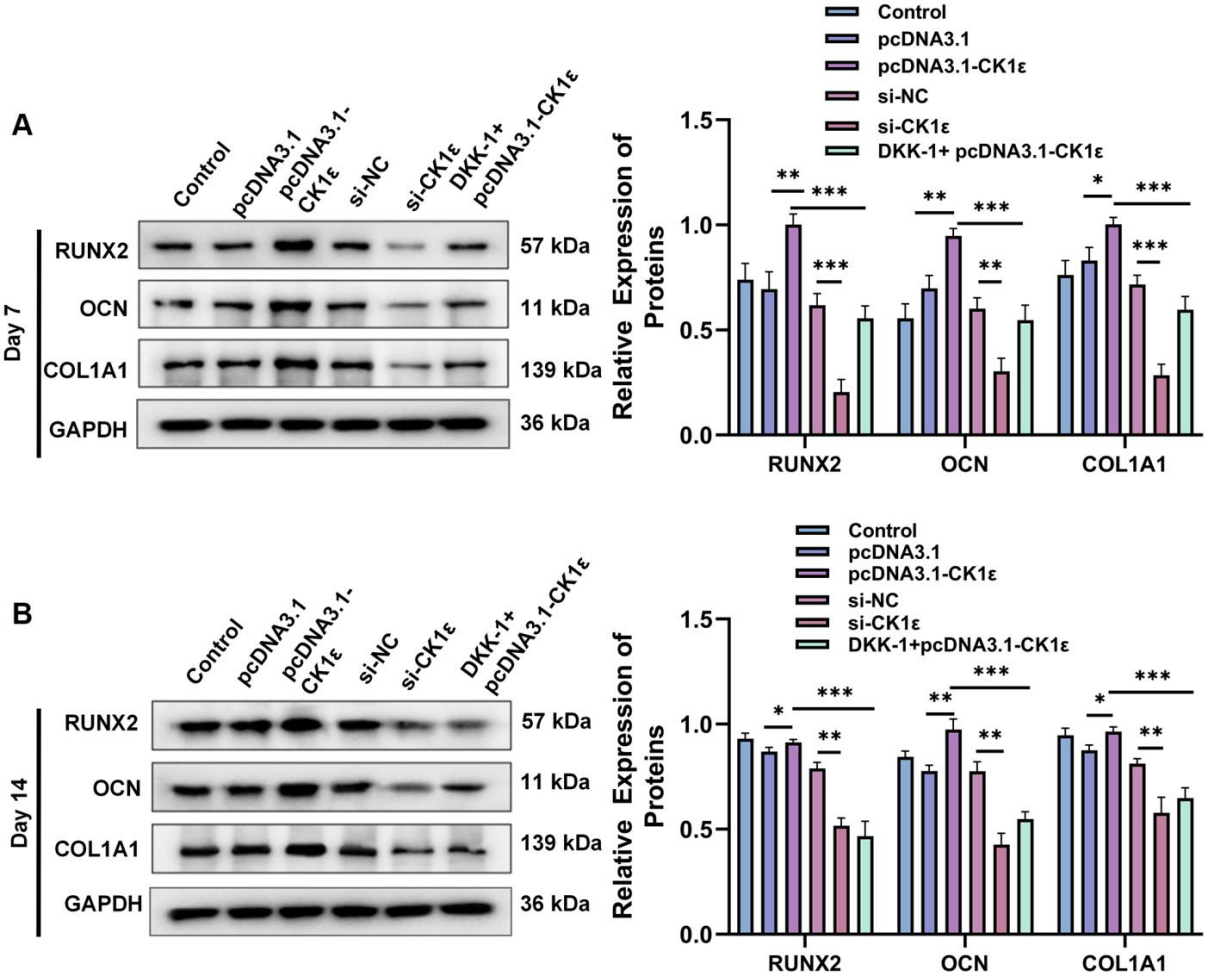
**Results of relative protein expression after OD of BMSC.** (**A**) Results of Western blot and relative protein expression levels of OD markers on the 7th day. (**B**) Results of Western blot and relative protein expression levels of OD markers on the 14th day. The experiments in this figure were repeated three times, and similar results were obtained. The data are presented as the means ± SD of independent experiments. The one-way ANOVA was used for (**A**, **B**). ^*^P < 0.05; ^**^P < 0.01; ^***^P < 0.001; Abbreviation: ns: not statistically significant.

We further verified the results of osteoblast-related protein expression between the different groups. At day 7, pcDNA3.1-CK1ε significantly increased the relative protein expression levels of the OD markers (RUNX2, OCN, and COL1A1) compared with the control group and the si-CK1ε group, while this trend was reversed by DKK-1 (Figure 5A). However, on day 14, the effect of DKK-1 on the OD marker protein still continued, while pcDNA3.1-CK1ε was weaker than that on day 7 (Figure 5B). We analyzed that the reason might be because at day 14, most of the BMSCs had completed OD. Moreover, similar results were observed in the mineralization assay and expression detection of marker proteins of osteogenic differentiation when DKK-1+pcDNA3.1-CK1ε group was compared with pcDNA3.1-CK1ε and si-CK1ε group. pcDNA3.1-CK1ε promotes osteogenic differentiation of BMSCs and is blocked by DKK-1, but this effect is weaker than that of si-CK1ε. The above results showed that transfection with pcDNA3.1-CK1ε promoted the OD of BMSCs, and the addition of DKK-1 significantly prevented the OD caused by CK1ε overexpression. CK1ε plays an important role in the Wnt signaling pathway. These results together indicated that the up-regulation of CK1ε could increase the OD of BMSCs by activating the Wnt/β-catenin pathway, considering that DKK-1 is an inhibitor of the Wnt signaling pathway co-receptor LRP5/6 [45]. Our results suggested that the target of CK1ε may be at the Wnt signaling pathway LRP5/6 receptor or at further downstream sites.

### CK1ε acts on DVL2 protein to activate Wnt/β-catenin signaling pathway and promote OD

**Figure 6:**
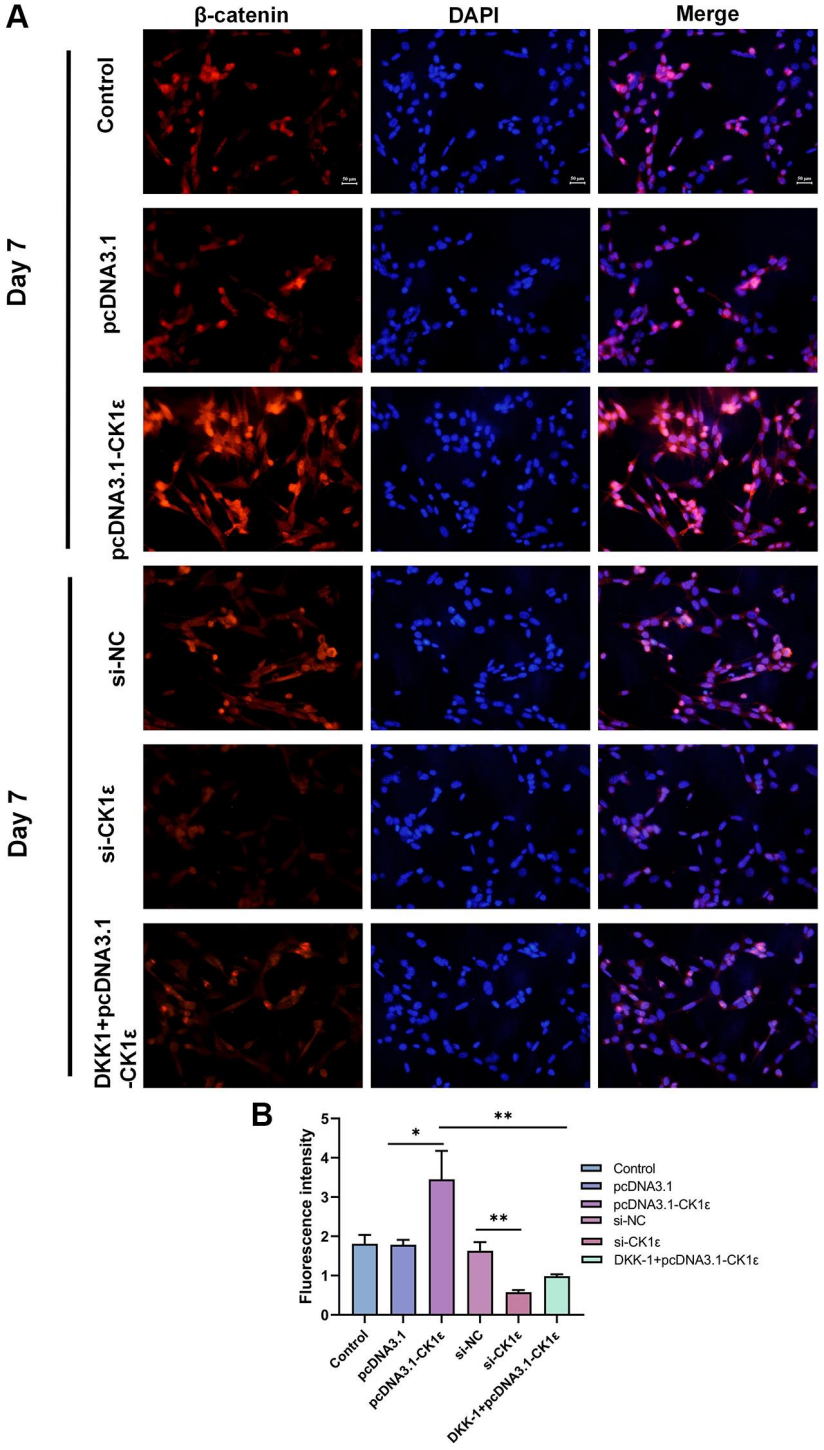
(**A**) Fluorescence microscopy images of β-catenin in BMSCs with different transfections on the 7th day and (**B**) relative fluorescence intensity analysis on the 7th day. The experiments in this figure were repeated three times, and similar results were obtained. The data are presented as the means ± SD of independent experiments. The one-way ANOVA was used for B. ^*^P < 0.05; ^**^P < 0.01; ^***^P < 0.001; Abbreviation: ns: not statistically significant.

In order to further explore the relationship between CK1ε and Wnt signaling pathway, we conducted follow-up experiments. The stimulation of the Wnt/β-catenin pathway by CK1ε was further observed by immunofluorescence staining on the seventh day after induction, and the results showed that CK1ε promoted the expression of β-catenin. Figure 6 shows the fluorescence microscope images and relative fluorescence intensity analysis of BMSCs in the different groups on day 7. Immunofluorescence staining showed that the expression of β-catenin in the nucleus of the si-CK1ε group was significantly lower than in the si-NC group. However, compared to the control group and the pcDNA3.1 group, BMSCs in the pcDNA3.1-CK1ε group showed higher relative β-catenin expression, suggesting that CK1ε up-regulation may promote OD by directly enhancing β-catenin expression. Next, the β-catenin inhibitor DKK-1 was added to the pcDNA3.1-CK1ε group to verify whether CK1ε gene overexpression could activate the Wnt/β-catenin pathway and increase the expression of β-catenin. After the DKK-1 intervention, the expression of β-catenin was decreased compared to the pcDNA3.1-CK1ε-transfected group, which indirectly confirmed that the up-regulation of CK1ε promoted the expression of β-catenin in BMSCs (Figure 6A, 6B). Based on the above results, we believed that the target of CK1ε may be located between the LRP5/6 receptor and β-catenin.

**Figure 7:**
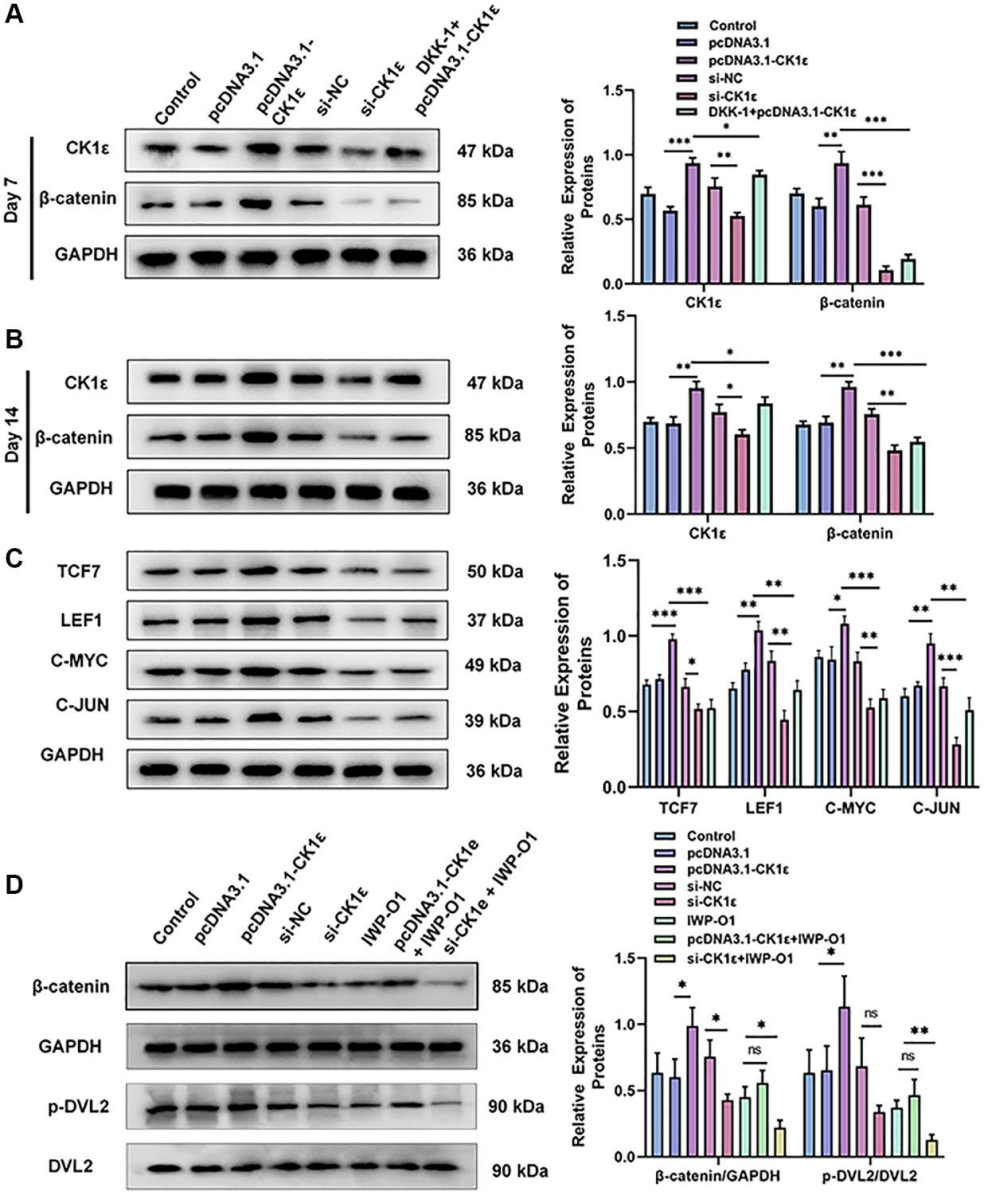
**Relative protein expression levels of related molecules related to the Wnt/β-catenin pathway.** (**A**, **B**) Results of Western blot and relative protein expression levels of CK1ε and β-catenin on the 7th and 14th days. (**C**) Results of Western blot and relative protein expression levels of downstream proteins related to the Wnt/β-catenin pathway. (**D**) Results of Western blot and relative protein expression levels of β-catenin, p-DVL2, and DVL2. The experiments in this figure were repeated three times, and similar results were obtained. The data are presented as the means ± SD of the independent experiments. The one-way ANOVA was used for (**A**–**D**). ^*^P < 0.05; ^**^P < 0.01; ^***^P < 0.001; Abbreviation: ns: not statistically significant.

We further verified the relative protein expression of CK1ε and β-catenin on day 7 and 14 after BMSC osteogenic induction by Western blots. At the same time, the relative protein expression changes of C-JUN, C-MYC, LEF1, and TCF7 in the downstream of Wnt/β-catenin pathway at day 14 were also verified. Figure 7A, 7B show that pcDNA3.1-CK1ε significantly increased the relative protein expression of CK1ε and β-catenin, while DKK-1 also inhibited the relative protein expression of β-catenin, but had little effect on CK1ε. However, the inhibition of β-catenin by DKK-1 was stronger than that by si-CK1ε, and the inhibition decreased at 14 days. Meanwhile, the downstream proteins of Wnt, including C-JUN, C-MYC, LEF1, and TCF7 [19, 46], showed similar changes to β-catenin (Figure 7C, *P* < 0.05). These results further demonstrate that CK1ε can activate the Wnt/β-catenin pathway when overexpressed, and its target location is upstream of β-catenin. By competitively binding to the LRP5/6 receptor, DKK-1 blocks the down transmission of the Wnt signal and reduces the Wnt signal entering the cytoplasm normally, which may be the reason why the expression of intracellular CK1ε itself is not inhibited. To explore the relationship between CK1ε and specific targets in the upstream region of the Wnt/β-catenin signaling pathway, we introduced the DVL protein inhibitor IWP-O1 (MCE, USA). IWP-O1 can specifically inhibit Dvl2 phosphorylation in cells [47]. Our results showed that IWP-O1 could inhibit the expression of β-catenin and also lowered the increases in β-catenin induced by pcDNA3.1-CK1ε. When IWP-O1 synergistically interacted with si-CK1ε, the inhibitory effect of si-CK1ε on the Wnt pathway was enhanced (Figure 7D, *P* < 0.05). The previous experimental results combined with the competitive binding effect of DDK-1 on low-density lipoprotein receptor-related protein (LRP) and the direct effect of DVL blocker, suggested that CK1ε may activate the Wnt/β-catenin pathway through direct interaction with DVL2 and activation of DVL2 phosphorylation.

### CK1ε significantly accelerates the healing of femoral bone defects in rats

**Figure 8:**
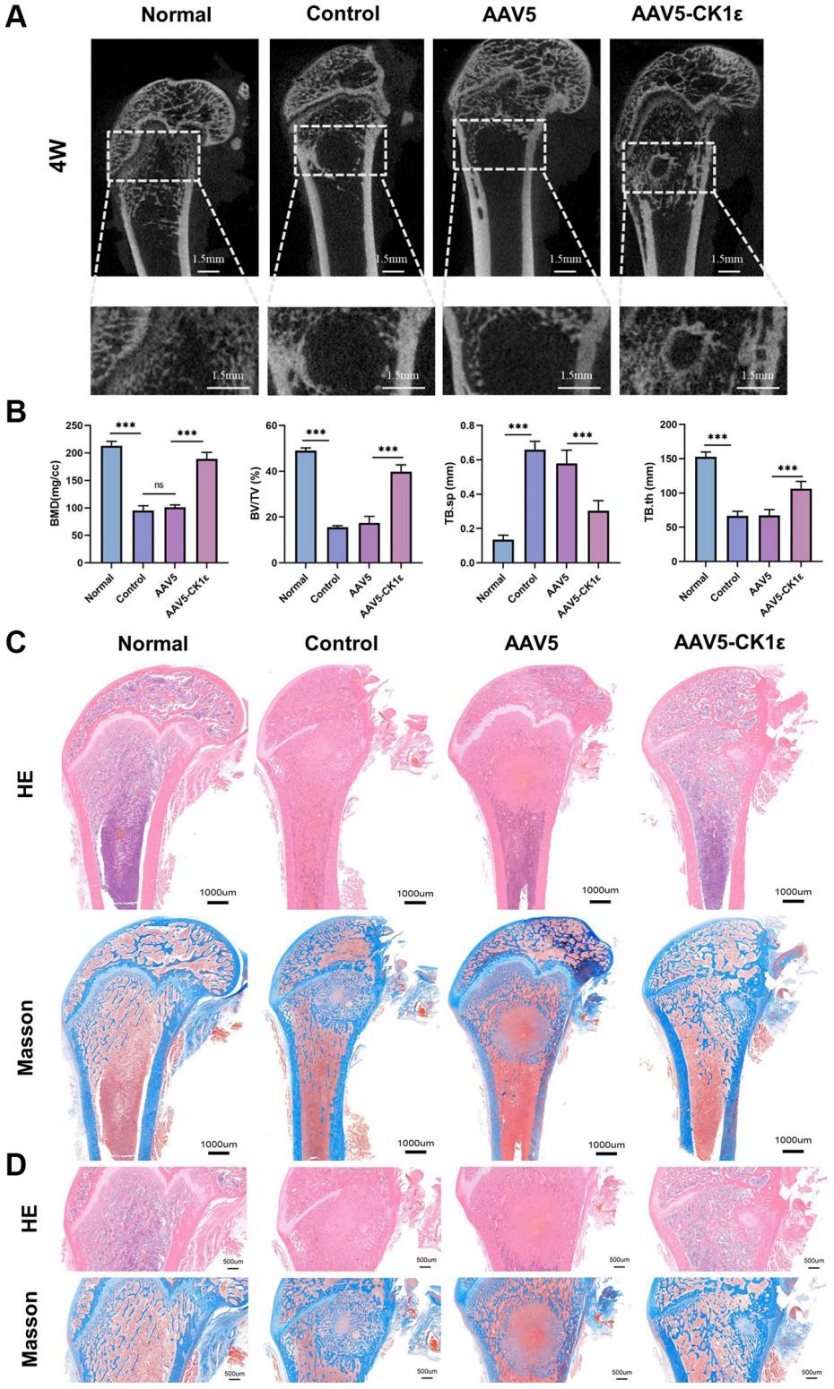
**New bone morphology and histological analysis of new bone formation.** (**A**) Micro-CT images of rats four weeks after surgery, and regional enlargement of the defect area. (**B**) Comparative quantitative and statistical analysis of BMD, BV/TV, TB.sp, and TB.th. (*n* = 5) (**C**). Defects of femoral condyle were stained with H&E and Masson’s stain four weeks after surgery. (**D**) Enlarged H&E and Masson’s stained images four weeks after surgery. The one-way ANOVA was used for B. ^*^P < 0.05; ^**^P < 0.01; ^***^P < 0.001; Abbreviation: ns: not statistically significant.

We established a rat model of a femoral condylar defect with a local injection to verify the effect of CK1ε intervention on bone defect healing in rats. The rats survived well and were killed 4 weeks after the operation. Specimens were collected for further experiments. First, Micro-CT was conducted, and histological images and data were collected for analysis. Bone defect reconstructions were the worst in the AAV5 and control groups (Figure 8A). The defect area was not healed and voids were seen in the femoral condyle area, which did not heal even four weeks after injury, indicating that the bone defect in the femoral condyle had a critical size (not self-healing). The tissue in the AAV5-CK1ε group gradually closed, showing complete tissue repair. At the same time point, we further analyzed the bone mass and density related indexes of the femoral condyle in rats. The results showed that compared with the control group and AAV5 group, the treatment of AAV5-CK1ε significantly increased the changes of BMD, BV/TV and TB.th, and decreased the changes of TB.sp (Figure 8B). These results suggest that AAV5-CK1ε could promote OD and be conducive to the effective formation of new bone tissue.

We obtained sagittal sections from the specimens collected at 4 weeks and performed H&E and Masson staining to analyze the effect of femoral condyle repair. Figure 8B shows the complete sagittal plane of the femur, and Figure 8C is a partial enlargement. From the gross images of H&E staining, we found that the bone tissue density in the AAV5-CK1ε and normal groups was significantly higher than that in the control and AAV5 groups, and that the AAV5-CK1ε group had the highest levels four weeks after surgery. Meanwhile, in both the control group and the AAV5 group, there were still traces of holes left over from the healing process (Figure 8C, 8D). Masson staining showed that in the original bone defect area of the AAV5-CK1ε group, there was obvious blue staining tissue infiltration, indicating collagen formation, and red staining with the surrounding collagen gradually tending to form normal cancellous bone tissue. It showed a good ability to promote OD. The H&E and Masson images of the AAV5-CK1ε group at 4 weeks showed that the bone defect had basically healed, and the cancellous bone area was no longer significantly different from normal bone tissue, suggesting that AAV5-CK1ε promoted bone defect healing (Figure 8C, 8D).

**Figure 9:**
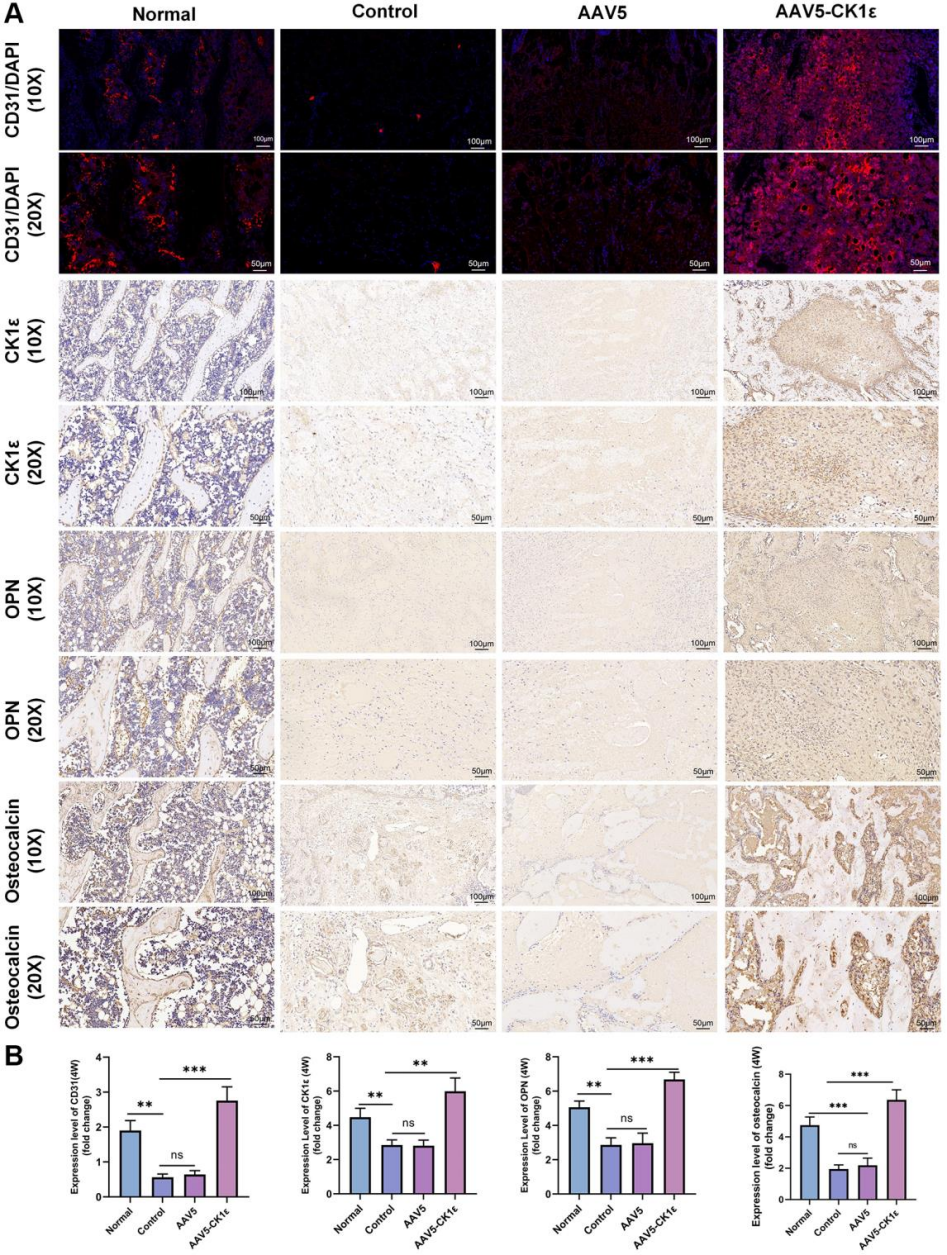
**Results of immunofluorescence and immunohistochemical staining and semi-quantitative analysis of femoral condylar defects.** (**A**) Images of CD31 immunofluorescence staining and immunohistochemistry of CK1ε, OPN, and osteocalcin 4 weeks after surgery. (**B**) Semi quantitative analysis results of immunofluorescence and immunohistochemistry (*n* = 5), using ImageJ software. The one-way ANOVA was used for B. ^*^P < 0.05; ^**^P < 0.01; ^***^P < 0.001; Abbreviation: ns: not statistically significant.

Secondly, the specimens were stained by immunohistochemistry. Immunohistochemical staining of CK1ε showed that compared with the AAV5 and control groups, the AAV5-CK1ε group was still significantly expressed *in vivo* at 4 weeks. The expression of osteoblast-related markers OPN and osteocalcin were verified (Figure 9A, 9B, *P* < 0.01). The signal of OPN and Osteocalcin in the AAV5-CK1ε group was significantly stronger than that in the control group and AAV5 group, and this result was verified by semi-quantitative analysis (Figure 9A, 9B, *P* < 0.01). Western blot analysis was used to detect CK1ε protein in samples from different groups, and the outcomes indicated that CK1ε relative protein expression in the AAV5-CK1ε group was significantly higher than that in the control and AAV5 groups (Supplementary Figure 3, *P* < 0.001). These results suggest that CK1ε can promote the healing process of femoral condylar defect in rats by accelerating the differentiation of osteoblasts.

### Vascularization of AAV5-CK1ε

The early angiogenesis and maturation of vascular sprouting are important driving factors for tissue repair. CD31, also known as a platelet-endothelial cell adhesion molecule, is an important indicator of angiogenesis. In this study, four weeks after injury, the CD31 signal in the AAV5-CK1ε group and the normal group was significantly stronger than that in the control group and the AAV5 group (Figure 9A). The quantitative analysis of CD31 expression was consistent with the immunofluorescence images (Figure 9B, *P* < 0.01). These results confirmed the mechanism of AAV5-CK1ε in promoting angiogenesis at the molecular level. *In vivo*, CK1ε has been shown to promote local angiogenesis, possibly through the Wnt/β-catenin signaling pathway. These results provide a good basis for understanding long-term bone tissue repair.

## DISCUSSION

**Figure 10:**
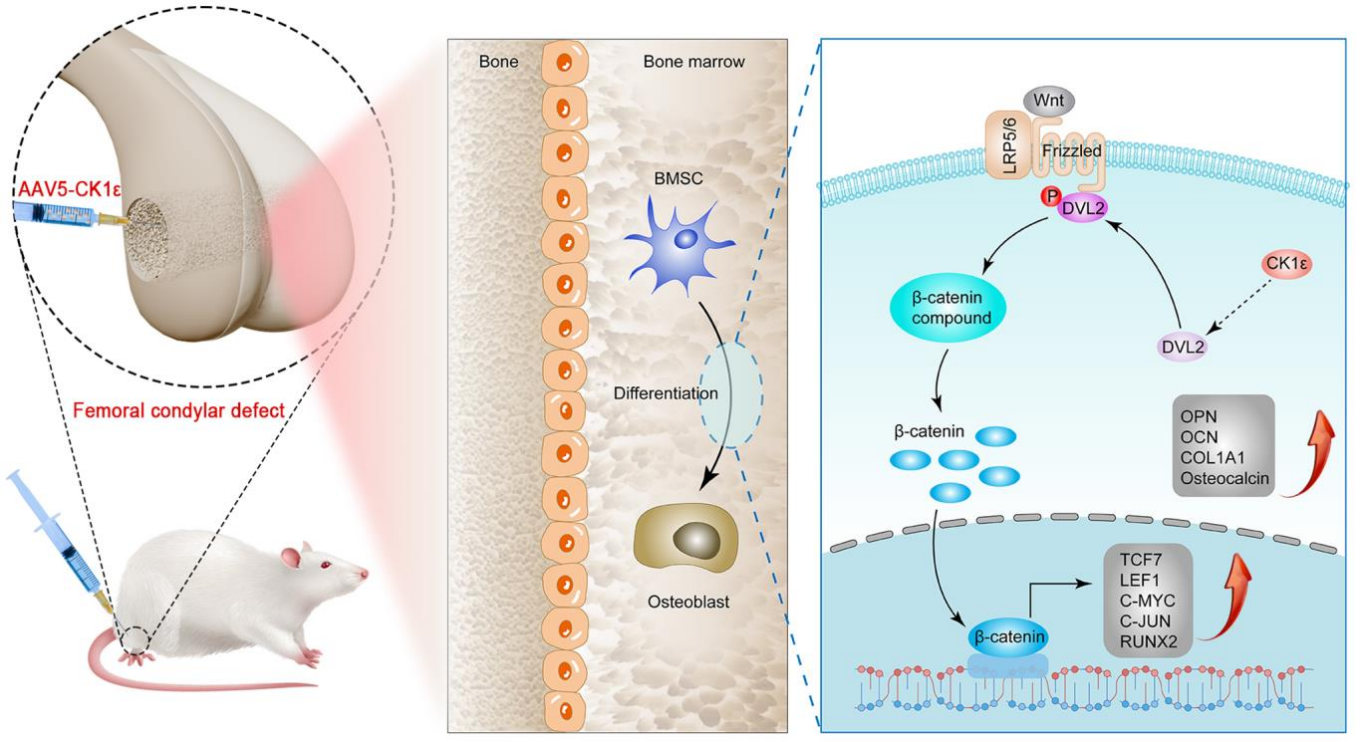
Schematic diagram of CK1ε promoting OD of BMSCs through activation of Wnt/β-catenin signaling, and repairing bone defects.

Tumor surgery, trauma, malformation, and infection are the main clinical problems leading to bone defects and also pose a huge challenge to bone reconstruction [1]. At present, in addition to standard treatments such as bone transplantation, the research on drugs and materials for bone regeneration and repair materials based on stem cells has also shown new vitality. BMSCs have a variety of differentiation tendencies in postures, including fibroblast-like morphology, adipogenesis, and osteogenesis, etc. BMSCs also have good proliferation, multiline differentiation, and immunomodulatory functions [48]. At the same time, the combination of MSC and biological scaffolds seems to be an ideal strategy for regenerative medicine [49]. The OD of osteoblasts derived from BMSCs is a complex and accurate regulatory process. Therefore, exploring the factors influencing the OD of BMSCs will help develop new strategies for the treatment of bone defects. Our *in vitro* study demonstrated that CK1ε intervention may promote BMSC OD through a variety of experimental methods, and this result may be achieved by activating DVL2 phosphorylation and thereby activating Wnt/β-catenin signaling. Further, *in vivo* studies have shown that CK1ε intervention has better efficiency of new bone formation in a femur condylar defect model, as well as enhanced local osteoblast action and angiogenesis (Figure 10).

CK1ε has been reported to be associated with a variety of diseases. A study by He et al. [50] showed that CK1ε was overexpressed in the articular cartilage of patients with osteoarthritis (OA), and OA development was promoted in experimental OA mice through the JNK pathway. In a study by Lin et al. [51], the low expression of CK1ε was associated with low survival rates in patients with liver cancer, suggesting that CK1ε may play a tumor suppressor role in hepatocarcinogenesis. Morgenstern [52] found that a lack of CK1ε could lead to the rapid clearance of intestinal stem cells, followed by growth arrest, recess villus atrophy, and the rapid death of mice. The first indication that CK1 family kinase regulates Wnt signaling is that injection of CK1ε mRNA into the ventral side of Xenopus embryos led to dorsalization and axial duplication similar to injection of Wnts [53]. At present, the relationship between the CK1 family and Wnt signaling pathway is still contradictory [24]. It was further shown that CK1ε forms a complex with Disheveled (Dvl) and Axin and positively regulates Wnt signaling by phosphorylating Dvl on multiple sites [53]. CK1ε and CK1γ mainly play a positive role in Wnt signaling through their influence on LRP6 and Dvl whereas CK1α mainly plays a negative role through its role in the regulation of β-catenin degradation, a genetic study in Drosophila also reveals a negative role for CK1ε and a positive role for CK1α in a genetically sensitized background [54]. Wnt activation was retained in all CK1ε-deficient intestinal cell populations except for Lgr5 + ISC, which showed DVL2-dependent Wnt signal attenuation. A study by Foldynová-Trantírková et al. [27] found that CK1ε was involved in mediating the Wnt/β-catenin pathway and played a regulatory role in the pathological process of breast cancer. Therefore, based on our previous research results, we proposed the hypothesis that CK1ε may be involved in mediating the OD of BMSCs and conducted a series of experiments to verify this hypothesis. We found that the expression level of CK1ε in the OD of BMSCs was higher than normal levels, suggesting that the up-regulation of CK1ε expression was associated with the OD of BMSCs. As an early marker of osteogenesis, ALP promotes mineralization during OD, and ARS indicates the number of calcium nodules in the late stage of OD [55]. Changes in markers of the OD of BMSCs, including RNX2, OCN, SP7, and COL1A1, further confirmed our conclusions. The effect of AAV5-CK1ε on bone repair in the rat model of femoral condylar defect also provides favorable evidence for our conclusion.

At present, the Wnt/β-catenin signaling pathway is one of the important mechanisms of OD and also the target signaling pathway of CK1ε. To further explore the relationship between CK1ε and Wnt/β-catenin, we introduced low and overexpressed CK1ε plasmids at the cell level and two key site-specific signaling pathway inhibitors of the Wnt pathway. The positive regulatory role of Wnt/β-catenin signaling pathway in the intervention of CK1 on the OD of BMSCs was preliminatively determined by the different expression of β-catenin under the intervention of pcDNA3.1-CK1ε and si-CK1ε. Therefore, we performed an in-depth investigation into whether CK1ε regulated OD by promoting Wnt/β-catenin signaling by detecting the expression level of Wnt/β-catenin. Our study showed that CK1ε overexpression significantly increased Wnt/β-catenin signaling-related genes. ALP activity, and the ARS and immunofluorescence results showed that DKK-1 could significantly prevent changes in these OD indicators. The seven-pass transmembrane receptor Frizzled (FZD) and single-pass low-density lipoprotein receptor-related protein 5 or 6 (LRP5/6) combine with Wnt ligands to form a Wnt–FZD–LRP5/6 trimeric complex [56], which inhibits the phosphorylation of β -catenin. Given the performance of CK1ε after DKK-1 intervention and β-catenin after CK1ε intervention, we hypothesized that the target of CK1ε in the Wnt/β-catenin signaling pathway lies between the β-catenin and LRP proteins. CK1ε may enable β-catenin to accumulate and localize in the nucleus, and then β-catenin binds to the transcription factor T cell factor/lymphoid enhancer factor (TCF/LEF), initiating downstream target genes. β-catenin phosphorylation by the kinase activity of CK1a and GSK3 is recognized by the E3 ubiquitin ligase subunit b-trcp, ubiquitinated, and transported to the proteasome for degradation to maintain low concentrations of β-catenin in the cells [57]. Qiong et al. [28] found that CK1ε could be activated by TGF-β1 in mouse precartilaginous stem cells, accompanied by the phosphorylation of GSK-3β, and the nuclear translocation of β-catenin. Nobutoshi Esaki et al. [58] found that Thr224 phosphorylation of DVL was required for the full activation of β-catenin transcriptional activity and that Daple overexpression induced CK1ε-mediated DVL2 phosphorylation at Thr224. After introducing the DVL2 phosphorylation inhibitor IWP-O1, we found that IWP-O1 alone inhibited pcDNA3.1-CK1ε-induced β-catenin expression and inhibited pcDNA3.1-CK1ε-induced β-catenin increases. The inhibitory effect of si-CK1ε on the Wnt pathway was enhanced when IWP-O1 acted synergistically with si-CK1ε. We also detected the downstream-related proteins of Wnt/β-catenin (C-JUN, C-MYC, LEF1, and TCF7) by immunoblotting to further study this regulatory pathway. The overexpression of CK1ε up-regulated all these proteins, suggesting that the up-regulation of CK1ε mediated by Wnt/β-catenin promoted OD. Therefore, we concluded that CK1ε might promote the OD of BMSCs by activating DLV2 phosphorylation, initiating Wnt signaling downstream, and activating β-catenin nuclear transfer. To further study the effect of CK1ε on bone loss in rats, the rat femoral condyle defect model was used [37, 38]. Micro-CT, immunohistochemical staining, and H&E and Masson’s staining analyses showed that CK1ε overexpression led to the rapid repair of bone defects and the increased expression of osteogenic marker genes.

However, our research had some limitations. As mentioned above, the Wnt/β-catenin signaling pathway is an important pathway in the OD of BMSCs, and after inhibiting this pathway, the role of CK1ε in promoting OD was significantly attenuated. However, we did not determine whether there are other signaling pathways or factors, as well as other cells in the local microenvironment, involved in the regulation of the OD of BMSCs, which requires further study. Secondly, a knockout animal model would bring a higher level of evidence to our animal model results. Once again, we did not conduct further research on animal locomotor ability and behavior.

## CONCLUSIONS

In summary, our results suggest that CK1ε can promote the OD of BMSCs by activating DVL2 phosphorylation and promoting β-catenin nuclear transfer, thus activating the β-catenin/Wnt signaling pathway. CK1ε may be a potential agent of choice for promoting bone defect healing. Overall, the importance of these findings is that they provide a potential strategy for treating bone defects and promoting bone regeneration.

## Supplementary Materials

Supplementary Figures
