# Supplementary material for: CK1ε drives osteogenic differentiation of bone marrow mesenchymal stem cells via activating Wnt/β-catenin pathway
Source: Aging (Albany NY). 2023 Oct 2;15(19):10193–212. doi: 10.18632/aging.205067 (PMC10599756; doi:10.18632/aging.205067)
Supplement: Supplementary Figures [file aging-15-205067-s001.pdf]

SUPPLEMENTARY FIGURES

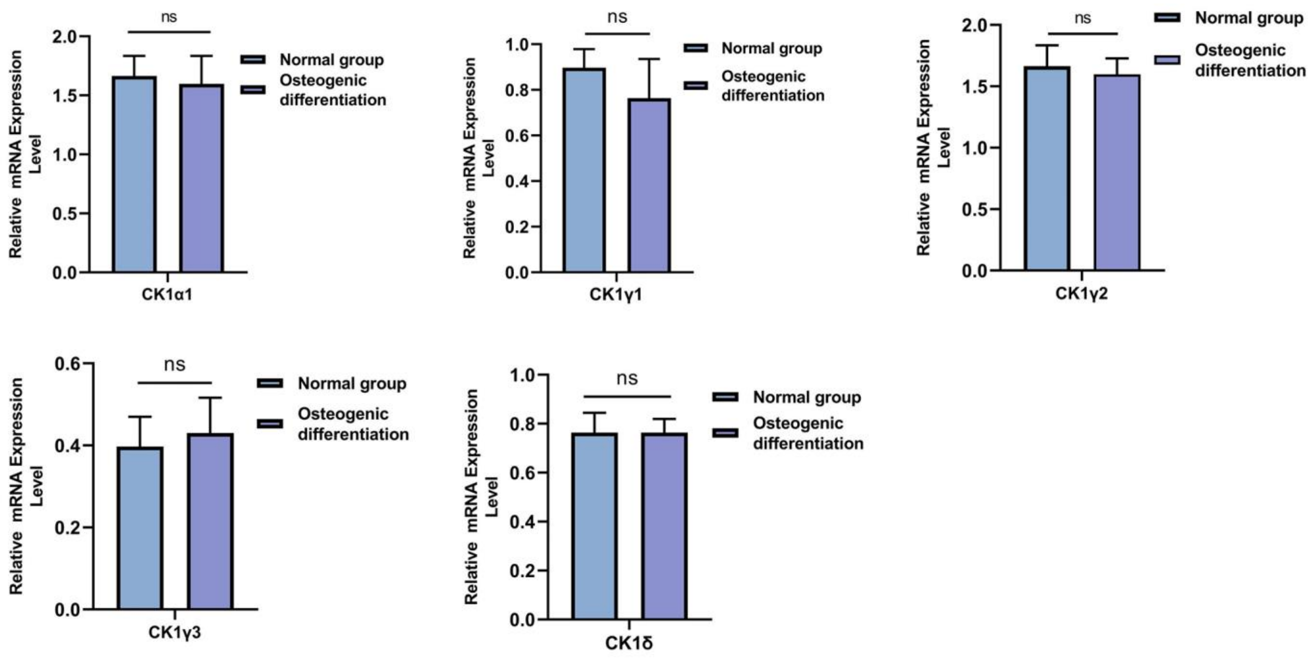

Supplementary Figure 1. The related mRNA expression levels of CK1 family members were detected by qRT-PCR analysis, after two weeks of incubation (*n* = 3). The experiments in this figure were repeated three times, and similar results were obtained. The student's *t*-test was used for statistical analysis. $^{*}P < 0.05$; $^{**}P < 0.01$; $^{***}P < 0.001$; Abbreviations: ns: not statistically significant.

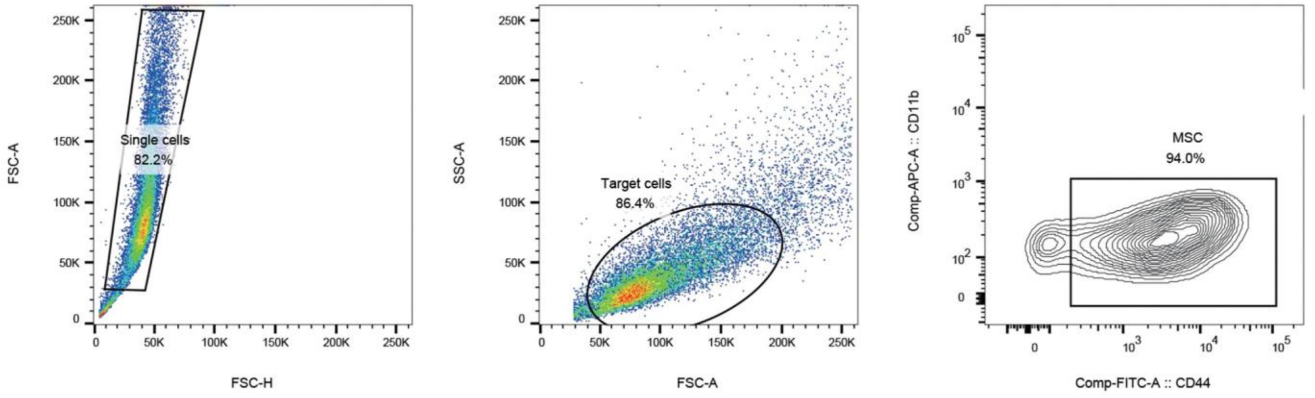

Supplementary Figure 2. Identification of rat BMSCs. CD44 and CD11b (biomarkers of OD) were examined by flow cytometry.

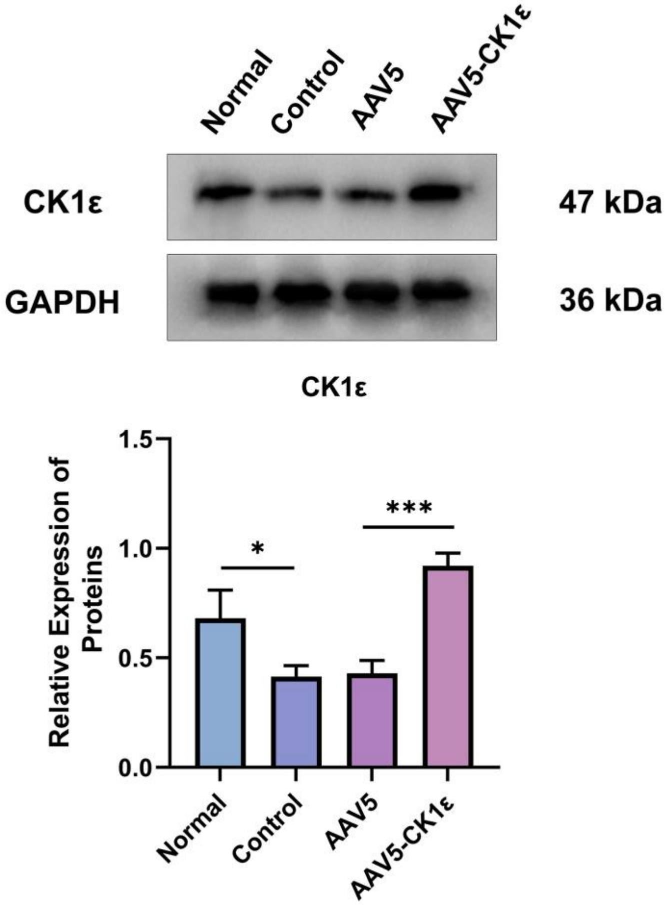

Supplementary Figure 3. CK1ε relative protein expression level and quantitative analysis (*n* = 5). The data are presented as the means ± SD of independent experiments. The one-way ANOVA was used. $^{*}P < 0.05$; $^{**}P < 0.01$; $^{***}P < 0.001$; Abbreviations: ns: not statistically significant.
